# Screening and characterization of β-galactosidase activity in lactic acid bacteria for the valorization of acid whey

**DOI:** 10.3168/jdsc.2021-0145

**Published:** 2021-11-25

**Authors:** Petar Kolev, Diana Rocha-Mendoza, Silvette Ruiz-Ramírez, Joana Ortega-Anaya, Rafael Jiménez-Flores, Israel García-Cano

**Affiliations:** Department of Food Science and Technology, The Ohio State University, 2015 Fyffe Ct., Columbus 43210

## Abstract

β-Galactosidase is an enzyme produced by some strains of lactic acid bacteria (LAB) commonly found in dairy products; however, industrial demand for these enzymes is still low. Acid whey (AW), a lactose-rich byproduct, has large output from cottage cheese and remains unexploited. The purpose of this study was to understand the production mechanism of β-galactosidase from LAB using AW as a culture medium. First, bioinformatics analysis was conducted on 15 species of LAB. Then, 24 strains were selected and inoculated in de Man, Rogosa, and Sharpe (MRS) broth and in AW medium to compare the bacterial kinetic growth and β-galactosidase production. Bacterial growth and total protein activity were measured using spectrophotometric techniques. β-Galactosidase activity was determined by 2 methods: following the hydrolysis of *o*-nitrophenyl-β-d-galactopyranoside and of 5-bromo-4-chloro-3-indoyl-β-d-galactopyranoside (X-gal) in tryptic soy agar plates. The relative expression of the β-galactosidase gene was performed using real-time quantitative PCR. Despite generally lower growth in AW, 18 strains showed higher β-galactosidase activity when grown in AW compared with MRS medium. The highest β-galactosidase activity in AW was in *Lactobacillus helveticus* strain OSU-PECh-4A, which showed almost 5 times higher activity than average. Analysis of 6 selected strains for expression of the *bgal-*620 gene found higher overexpression in AW than in MRS, regardless of specific β-galactosidase activity. Strains of LAB such as OSU-PECh-4A could valorize AW through the production of β-galactosidase (as an aid to lactose digestion) and production of prebiotic galactooligosaccharides.

## Highlights

• Lactic acid bacteria (LAB) have significant production of β-galactosidase despite low levels of cellular growth.
• Significantly lower lactose content of acid whey (AW) after LAB inoculation makes disposal more ecofriendly.
• *L. helveticus* strain OSU-PECh-4A displayed intense β-galactosidase activity when cultured in AW.
• To further understand the production of the β-galactosidase, quantitative PCR analysis should be used to evaluate real-time gene expression.

## Graphical Abstract

**Figure:**
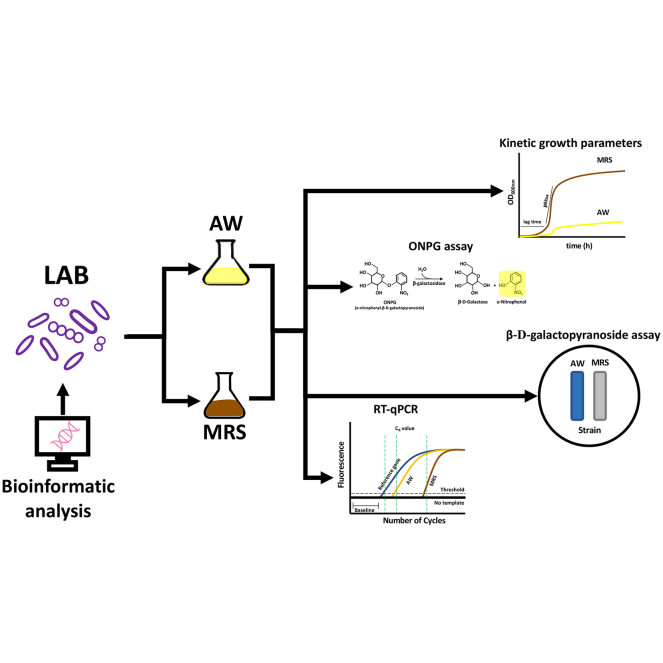
**Summary:** Bioinformatic analysis as the first screening of β-galactosidase activity of multiple species and strains of LAB provides valuable information for selecting strains with this activity. Following inoculation in acid whey (AW) and commercial medium (de Man, Rogosa, and Sharpe; MRS), kinetic growth parameters were determined. To find strains with high β-galactosidase activity, different substrates were used (o-nitrophenyl-β-d-galactopyranoside and β-d-galactopyranoside). LAB showed higher β-galactosidase activity when cultured in AW compared with commercial MRS medium; thus, AW secondary metabolites likely complement the upregulation to promote enzyme production. *Lactobacillus helveticus* OSU-PECh-4A was identified as a strain with exceptional β-galactosidase activity. The bgal-620 gene was upregulated in OSU-PECh-4A cultured in AW. This strain can valorize AW and be used to further study β-galactosidase.

This work focused on acid whey that is derived from cottage cheese production and recovered after the fermentation of skim milk for approximately 5 h (Kosikowski and Mistry, 1997). Whey has nutritional value because it retains approximately 55% of the nutrients in milk (Panesar et al., 2007). The 2 forms of whey are acid whey (**AW**), a byproduct of Greek yogurt and cottage cheese production, and sweet whey, a byproduct from the production of most cheeses. Acid whey has a lower pH than sweet whey: 4.2–4.8 compared with 6.0–7.0, respectively; it also has higher lactose and mineral contents (Rocha-Mendoza et al., 2021). The nutritional richness of AW has allowed its successful use in producing functional beverages, as a medium for the cultivation of microorganisms, among other uses (Rocha-Mendoza et al., 2021). One use that has the potential for industrial scaling and large market growth is the use of AW as a medium for β-galactosidase production (Bentahar et al., 2019).

β-Galactosidase, commonly known as lactase, is an enzyme that catalyzes the hydrolysis of the β-glycosidic bond between galactose and glucose (Juers et al., 2012). The enzyme is widespread in various microorganisms, including bacteria, fungi, and yeast (Saqib et al., 2017). A deficiency in the production of the enzyme in humans causes the condition known as lactose intolerance. In addition, β-galactosidase has the ability to catalyze the transgalactosylation of lactose into allolactose (Juers et al., 2012). Following allolactose synthesis, β-galactosidase can polymerize the disaccharide into galactooligosaccharides (**GOS**; Yañez-Ñeco et al., 2021). Galactooligosaccharides are considered prebiotics, and finding specific microorganisms that produce high yields of GOS is the topic of a large amount of contemporary research (Yañez-Ñeco et al., 2021; Zerva et al., 2021).

In lactose-rich environments, β-galactosidase can be effectively obtained from multiple microorganisms including microalgae (Bentahar et al., 2019) and lactic acid bacteria (**LAB**), the latter of which have been used previously to produce β-galactosidase (Carevic et al., 2015) from skim milk and supplemented sweet whey (Vasiljevic and Jelen, 2001). Supplemented AW could provide a more hospitable environment for LAB than sweet whey, because of its higher vitamin, mineral, and lactose concentrations and, presumably, the presence of secondary metabolites produced by starter cultures during the long fermentation of cottage cheese. Typically, the supplementation is carried out with yeast extract, which has a high protein content (Rocha-Mendoza et al., 2021) that is important for LAB, as nitrogen is crucial for their growth.

In this study, we first investigated the growth of LAB in supplemented AW compared with de Man, Rogosa, and Sharpe (**MRS**) broth, an optimal medium for LAB growth that contains no lactose. The subsequent objectives were to determine whether the β-galactosidase activity of LAB cultivated in AW was significantly higher than that of LAB cultivated in MRS and to identify specific strains with exceptional β-galactosidase activity, as well as to evaluate expression of the gene encoding this protein. The strains studied in this work have the potential for use in industrial-scale production of AW for many purposes, including production of β-galactosidase for the lactose-intolerant market, utilization of β-galactosidase for the novel use of GOS production, or to reduce the biological oxygen demand of AW before disposal.

In this study, we used 137 strains of LAB from the Ohio State University Parker Endowed Chair (OSU-PECh) collection. The strains were isolated from a variety of dairy sources and were stored at −80°C in cryovials containing MRS (BD Difco) broth and glycerol (80%/20%; García-Cano et al., 2019). *Staphylococcus epidermidis* ATCC 1222 was used as a negative control and cultivated in brain heart infusion broth (BD Difco) and incubated at 37°C. The selection of specific LAB strains for screening was determined following bioinformatics analysis using the tools available from the National Center for Biotechnology Information (NCBI, https://www.ncbi.nlm.nih.gov/), which also allowed for the identification of genes (Pruitt et al., 2007), specifically the *bgal-*620 gene encoding β-galactosidase.

The AW used in this work was obtained from Superior Dairy (Canton, OH) as a byproduct of cottage cheese production. To remove sediment and potential contaminants, the AW was centrifuged (Sorvall Legend XF, Thermo Scientific) at 13,664 × *g* at 4°C for 25 min, enriched with 0.5% final concentration of yeast extract (Sigma-Aldrich), and autoclaved before inoculation.

To screen the initial β-galactosidase activity of the bioinformatically selected strains, tryptic soy agar (Remel) plates were prepared, and 40 mL of 5-bromo-4-chloro-3-indoyl-β-d-galactopyranoside (X-gal 20 mg/mL, ThermoFisher Scientific) was added to each plate as a substrate and spread evenly (β-d-galactopyranoside test). The plates were dried for 30 min and then streaked with the MRS and AW cultures. A positive enzymatic result was indicated by the observation of a blue color surrounding the growth of the colonies, which is caused by cleavage of the β-d-galactopyranoside bond by β-galactosidase, releasing the blue color of the indole.

Ten microliters of each strain found to have β-galactosidase activity was inoculated in 3 mL of MRS broth and incubated at 37°C for 16 h. Following incubation, 50 mL of the broth was used to further inoculate one culture tube containing 3 mL of MRS broth and another containing 3 mL of AW. Then, 200 mL of the inoculated AW and MRS broth was transferred into separate wells within a sterile 96-well flat-bottomed plate (Corning Inc.). Growth curves were obtained by measuring the optical density at 600 nm (**OD_600_**) in a microplate reader (Multiskan GO, ThermoFisher Scientific) with absorbance readings taken at 30-min intervals for 16 h at 37°C (Rocha-Mendoza et al., 2020).

Next, 1.5 mL of each AW and MRS culture was centrifuged at 16,000 × *g* at 4°C for 10 min (Centrifuge 5424, Eppendorf). The cell pellets were washed and resuspended in Tris-HCl buffer (50 m*M*, pH 7.6). The resuspended cells were diluted to an OD_600_ of 5.00, placed on ice, and sonicated (Branson Ultrasonics Corp.) at 30 Hz for 7 min. The sonicated solution was centrifuged at 21,300 × *g* at 4°C for 10 min to eliminate cell debris and unbroken cells. The supernatant or protein extract was used for further assessment of enzymatic activity.

The total protein content was measured in the supernatant by transferring 1 mL of the solutions into a 1-mL high-precision quartz cuvette (Hellma Analytics), which blocks spectrophotometric interference, and the absorbance at 280 nm was measured. These readings were used to determine total protein content by using a standard curve prepared from known concentrations of BSA (Pierce/ThermoFisher Scientific).

The β-galactosidase activity in the protein extracts was determined using a modified procedure described by Bentahar et al. (2019). The method uses *o*-nitrophenyl-β-d-galactopyranoside (**ONPG**) to determine β-galactosidase activity. The ONPG molecule is colorless, but upon contact with β-galactosidase, it is cleaved to form galactose, which is colorless, and *o*-nitrophenyl, which is yellow. A 10 mg/mL solution of ONPG (Sigma-Aldrich) was prepared, and a 96-well plate was arranged with each well containing 100 mL of protein extract and 100 mL of ONPG solution. The blank was prepared using 100 mL of Tris-HCl (50 m*M*, pH = 7.8) buffer and 100 mL of ONPG solution. The samples were then incubated for 12 h at 37°C. Following incubation, 50 mL of 140 m*M* of Na_2_CO_3_ was added to each well of the plate to stop the reaction. Then, absorbance at 420 nm was measured. One unit (U) of β-galactosidase activity was defined as the change of 0.001 absorbance units (U) per min (U/min). The specific activity was correlated with the protein concentration (U/min × mg of protein).

In addition, LAB strains were cultured in AW and MRS broth. The cell pellet was recovered and washed as described previously. RNA and cDNA extraction followed the procedure described in Rocha-Mendoza et al. (2020) using the RNeasy Plus Mini Kit (Qiagen) according to the manufacturer's instructions. A PCR was performed using the materials and instructions of the iScript RT kit (Bio-Rad) to synthesize cDNA from 1 mg of RNA. The extracted cDNA concentration was adjusted to 50 ng/mL and stored at −80°C.

The expression of *bgal-*620 in each strain was measured using quantitative PCR methods described in Rocha-Mendoza et al. (2020). The primers were designed based on bioinformatics analysis using a recurring AA sequence that 93.8% of the LAB have present. We named this gene *bgal-*620 and the primers used were as follows: 620F: 5′-GATCGCCACTCCGATTATGAA-3′ and 620R: 5′-AGCCATAATAATATCTCACCTCCTG-3′. Real-time PCR was performed using iTaq Universal SYBR Green Supermix (Bio-Rad) in a C1000 Touch Thermal Cycler (Bio-Rad). Expression of the *bgal-*620 gene was normalized using the reference gene (16S rRNA). Analysis was performed using CFX Maestro Software 3.1 (Bio-Rad), under the ΔΔCq method, where Cq is the cycle threshold (CFX Maestro software user guide, version 1.1, 2017; Bio-Rad). Each experiment was performed in triplicate, and one-way ANOVA was run using JMP Pro 14 software (SAS Institute Inc.) to determine significant differences.

Table 1 shows the 3 key parameters obtained from the kinetic growth curves of the LAB: rate of bacterial growth (**μMax**); change in optical density at 600 nm (**ΔOD_600nm_**), which indicates the total amount of bacterial growth; and lag time, which is the period during which bacteria adapt to their environment before beginning rapid cell division.

**Table 1:** Key parameters of kinetic growth^1^ of selected stains of lactic acid bacteria

Strain^2^	AW medium			MRS medium		
	μMax (h^−1^)	Change in OD	Lag time (h)	μMax (h^−1^)	Change in OD	Lag time (h)
*Lactobacillus acidophilus* OSU-PECh-1A	0.08	0.11	3.88	0.11	1.24	5.3
*Lactobacillus helveticus* OSU-PECh-1B	0.11	0.15	3.32	0.12	1.24	4.79
*Lacticaseibacillus casei* OSU-PECh-2	0.14	0.34	35.22	0.09	1.51	9.17
*Lactobacillus helveticus* OSU-PECh-4A	0.05	0.55	10.32	0.06	1.18	6.59
*Lactobacillus acidophilus* OSU-PECh-5	0.15	0.33	12.56	0.05	0.45	3.14
*Lactobacillus gasseri* OSU-PECh-6B	0.13	0.18	7.73	0.14	1.31	5.15
*Lactiplantibacillus pentosus* OSU-PECh-6C	0.1	0.15	3.88	0.08	0.92	3.89
*Lacticaseibacillus rhamnosus* OSU-PECh-8A	0.12	0.23	4.59	0.14	1.35	10.18
*Lacticaseibacillus paracasei* OSU-PECh-8B	0.12	0.2	4.11	0.13	1.35	8.49
*Lactobacillus johnsonii* OSU-PECh-9	0.11	0.21	5.35	0.13	0.18	1.9
*Lactobacillus gasseri* OSU-PECh-10A	0.12	0.21	4.11	0.15	0.49	2.35
*Lacticaseibacillus paracasei* OSU-PECh-10B	0.13	0.21	3.73	0.14	0.5	2.39
*Lacticaseibacillus casei* OSU-PECh-11A	0.12	0.26	5.83	0.13	1.39	4.21
*Lactobacillus crispatus* OSU-PECh-11B	0.15	0.5	8.84	0.15	1.39	4.55
*Pediococcus pentosaceus* OSU-PECh-13	0.11	0.15	4.28	0.1	0.72	4.99
*Lactobacillus amylovorus* OSU-PECh-16	0.14	0.29	4.25	0.12	0.18	1.61
*Enterococcus faecium* OSU-PECh-27A	0.06	0.11	9.47	0.03	0.41	2.86
*Lactobacillus amylovorus* OSU-PECh-32	0.16	0.24	6.25	0.1	1.42	4.73
*Enterococcus mundtii* OSU-PECh-39B	0.06	0.32	15.68	0.04	1.86	9.73
*Lactobacillus crispatus* OSU-PECh-40B	0.16	0.41	9.03	0.21	1.39	3.36
*Limosilactobacillus reuteri* OSU-PECh-48	0.11	0.25	3.69	0.13	1.28	4.78
*Limosilactobacillus reuteri* OSU-PECh-66B	0.03	0.04	4.81	0.04	0.78	6.56
*Pediococcus acidilactici* OSU-PECh-74	0.18	0.58	8.59	0.16	1.37	4.28
*Lactobacillus delbrueckii*OSU-PECh-75	0.12	0.21	3.16	0.14	0.31	2.02

1 AW = acid whey; MRS = de Man, Rogosa, and Sharpe; μMax = maximum growth rate; OD = optical density.

2 OSU-PECh refers to strains in the Ohio State University Parker Endowed Chair collection.

As expected, LAB cultured in MRS recorded significantly higher ΔOD_600nm_, higher μMax values, and shorter lag times compared with LAB cultured in AW because of the rich composition of MRS broth, which includes peptone, beef extract, yeast extract, and glucose, providing sources of carbohydrates, nitrogen, vitamins, and minerals, making it an excellent medium for LAB.

Stark differences in ΔOD_600nm_ for LAB cultured in MRS and AW were observed in *Lactobacillus acidophilus* OSU-PECh-1A, *Lacticaseibacillus casei* OSU-PECh-11A, and *Enterococcus mundtii* OSU-PECh-39B. Another factor that can influence growth is the pH of the medium. The initial pH of AW was 4.3 and that for MRS was 7.0. It is not surprising that the LAB used in this study have the ability to grow at acidic pH. Different LAB strains have been reported for their ability to grow at low pH (pH 4.3), indicating greater adaptation or sensitivity to pH (Rocha-Mendoza et al., 2020).

The longer lag times observed for LAB cultured in AW compared with MRS were likely due to the differences in carbohydrate content between the 2 media. The sole carbon source in MRS is glucose, which is readily fermentable by LAB, whereas the primary carbon source in AW is lactose, which necessitates the production of β-galactosidase for utilization. The lag times for *L. helveticus* OSU-PECh-4A cultured in AW and MRS had a greater than average difference, 10.32 and 6.59 h, respectively (*P* < 0.05). It is likely that this lag time is due to β-galactosidase production: Figure 1A shows that the specific β-galactosidase activity of OSU-PECh-4A cultured in AW was more than twice as high (*P* < 0.05) as the activity of this strain cultured in MRS.

**Figure 1:**
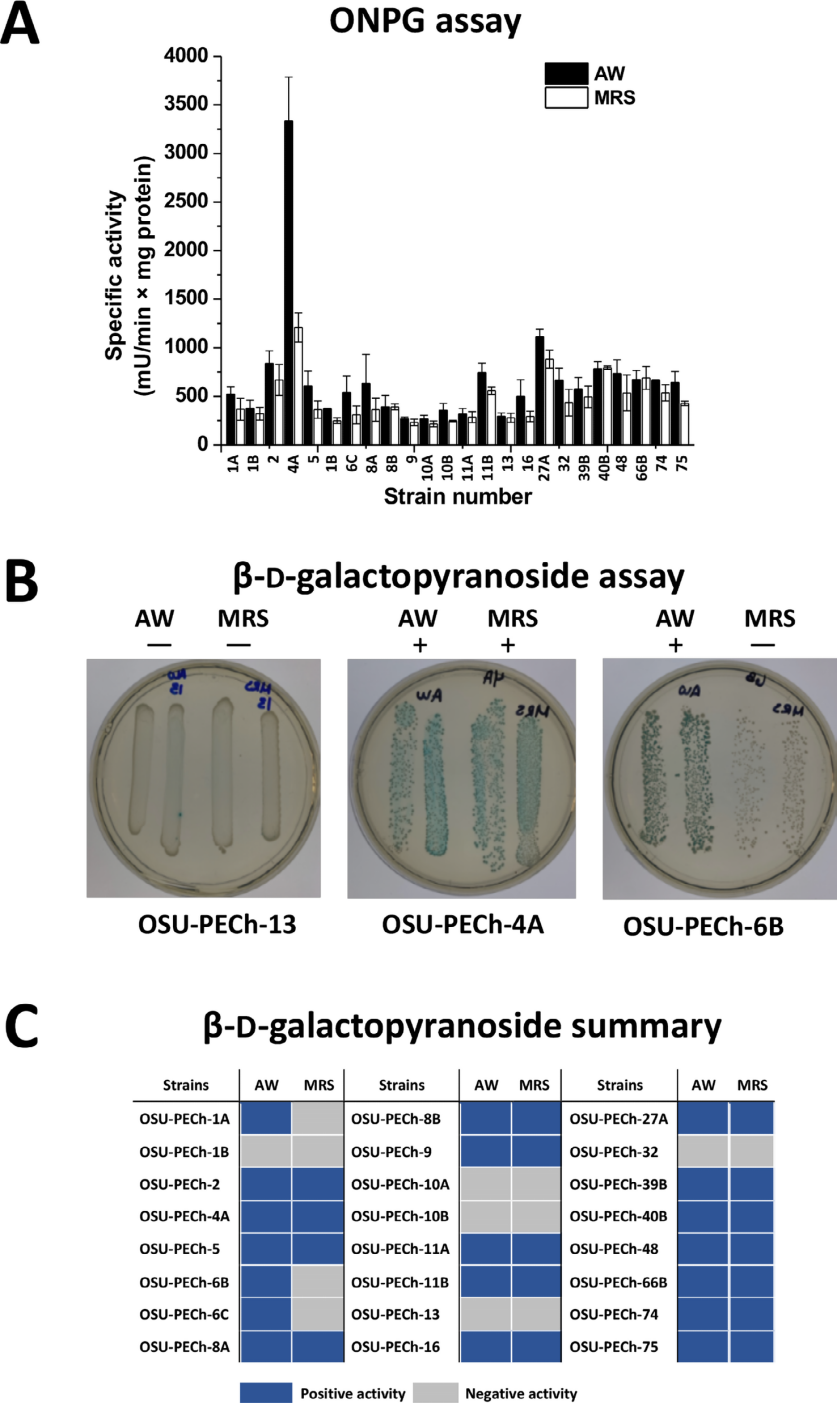
(A) The specific β-galactosidase activity of proteins extracted from 24 strains of lactic acid bacteria (LAB) when cultured in acid whey (AW) and de Man, Rogosa, and Sharpe (MRS) broth, using *o*-nitrophenyl-β-d-galactopyranoside (ONPG) as a substrate. Error bars indicate the SD of 3 independent experiments. (B) Examples of positive and negative results of an β-d-galactopyranoside test. (C) Results of β-d-galactopyranoside test performed on selected strains of LAB. OSU-PECh refers to strains in the Ohio State University Parker Endowed Chair collection.

Figure 1A shows that 83.3% of the 18 LAB strains displayed higher specific β-galactosidase activity in AW than in MRS; the 16.7% of strains that displayed higher activity in MRS differed only slightly between media. These results demonstrate that the difference between the carbon sources of the 2 media had a significant effect on the degree of β-galactosidase activity, whereas greater and faster growth in culture, indicated by higher values of ΔOD_600nm_ and μMax, had little effect on β-galactosidase activity.

The LAB with the highest specific β-galactosidase activity in AW were *L. helveticus* OSU-PECh-4A, and *Enterococcus faecium* OSU-PECh-27A, with specific activity of 3,331, and 1,110 mU/min × mg protein, respectively. These strains displayed exceptional β-galactosidase activity in both AW and MRS, but their activity in AW was significantly higher (*P* < 0.05), making the strains ideal for β-galactosidase production, as greater β-galactosidase activity valorizes AW nutritionally and economically through synthesis of GOS (Yañez-Ñeco et al., 2021).

The β-d-galactopyranoside test was performed to confirm the specific β-galactosidase activity reported, as *Staphylococcus* species have been shown to provide false-positive results for the ONPG test (Tse et al., 2012), indicating the potential risk of false positives from other bacteria. Examples of the characteristic blue color of bacterial colonies on β-d-galactopyranoside plates, differentiating bacteria that produce β-galactosidase from those that do not, are shown in Figure 1B. *Staphylococcus epidermidis* was used as a negative control; it does not have β-galactosidase activity (Becker and von Eiff, 2011).

The results of the β-d-galactopyranoside test (Figure 1C) conflicted with the results of the bioinformatics analysis, as all 24 strains selected were reported to contain β-galactosidase genes, but 5 strains did not show the ability to hydrolyze the X-gal substrate, which means that the gene was not expressed in the conditions tested. Conversely, *Lactobacillus delbrueckii* ssp. *lactis* OSU-PECh-75 was reported to lack β-galactosidase genes and it did have a positive result. Although not reported in Figure 1A, *L. helveticus* OSU-PECh-1B and *Lactobacillus amylovorus* OSU-PECh-32 both displayed moderate β-galactosidase activity in AW. It was notable that these strains tested negative to the β-d-galactopyranoside test because a strain from each species tested positive: *L. helveticus* OSU-PECh-4A and *Lactobacillus amylovorus* OSU-PECh-16. These results indicate either large variance in β-galactosidase production within LAB species or potential shortcomings of the β-d-galactopyranoside method.

The bioinformatics analysis discovered a recurring gene in 93.8% of the LAB species selected in this study, between 625 and 644 AA in length, that we referred to as the *bgal-*620 gene. For the real-time quantitative PCR, 6 strains were selected, 2 with a negative result to the β-d-galactopyranoside test (*L. helveticus* OSU-PECh-1B and *Lacticaseibacillus paracasei* OSU-PECh-10B), 2 with moderate β-galactosidase activity (*L. acidophilus* OSU-PECh-1A and *Lactiplantibacillus pentosus* OSU-PECh-6C), and 2 with high β-galactosidase activity (*L. helveticus* OSU-PECh-4A and *E. faecium* OSU-PECh-27A). From Figure 2, it can be seen that all strains displayed higher gene expression when cultured in AW than in MRS, which corresponds to the greater production of β-galactosidase–encoding mRNA. For OSU-PECh-4A, OSU-PECh-6C, and OSU-PECh-27A, respectively, the expression was more than 3.3, 34, and 4 times greater when grown in AW medium than in MRS. Both OSU-PECh-1B and OSU-PECh-10B tested negative to the β-d-galactopyranoside test (Figure 1C); however, only OSU-PECh-10B displayed significant gene expression in AW. OSU-PECh-4A, which displayed the highest specific β-galactosidase activity using ONPG substrate, did not display the highest gene expression; instead, OSU-PECh-6C (moderate activity) showed the highest gene expression, closely followed by OSU-PECh-27A. It should be noted that the correlation between the content of a specific protein and mRNA specific to the protein is only about 40% (Vogel and Marcotte, 2012). Furthermore, in this experiment, the β-galactosidase content was measured indirectly through its specific activity, which results in a low correlation with the gene expression. Therefore, the strain with the highest specific β-galactosidase activity, OSU-PECh-4A, was not necessarily the strain with the highest gene expression. The one-way ANOVA tests determined that the differences in expression of *bgal*-620 in AW among the strains tested was significant (except for OSU-PECh-1B). These results imply that the environment that AW provides for LAB, where lactose is the sole carbon source, caused significant overexpression of the β-galactosidase gene, further showing that AW is an ideal medium for enzyme production.

**Figure 2:**
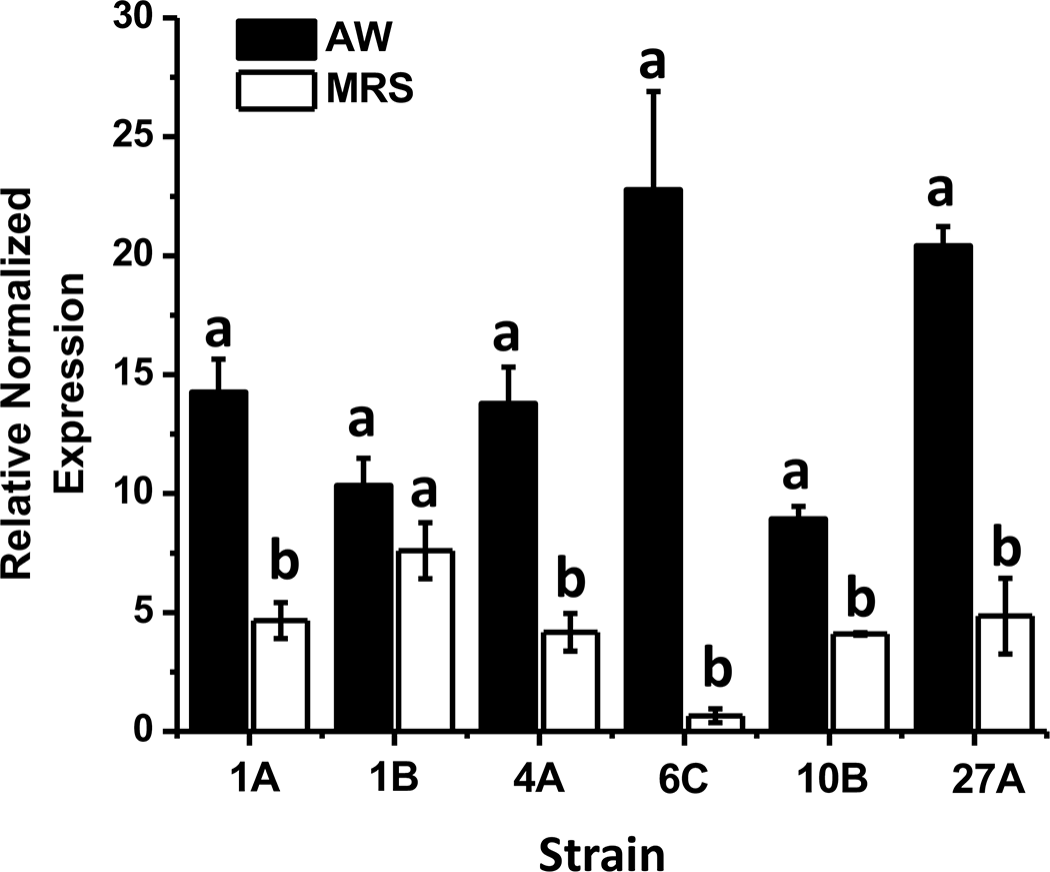
Relative gene expression of *bgal-*620 encoding β-galactosidase production in various lactic acid bacteria (LAB) cultured in acid whey (AW; filled bars) and de Man, Rogosa, and Sharpe (MRS; empty bars) broth. Error bars represent the mean ± SD of 6 independent experiments. Strains are defined in Table 1. Different lowercase letters (a, b) indicate significant difference (*P* < 0.05).

Overall, 83.33% of LAB strains tested displayed higher specific β-galactosidase activity in AW than in MRS broth. Although this is to be expected, it was an unusually high response to production of this enzyme by some strains. The strains *L. helveticus* OSU-PECh-4A and *E. faecium* OSU-PECh-27A displayed exceptional specific β-galactosidase activity in AW. The β-d-galactopyranoside test confirmed the β-galactosidase activity of most strains selected by the bioinformatics analysis. Finally, we showed that all strains had higher expression of *bgal-*620 (encoding β-galactosidase) in AW compared with MRS, regardless of the results of the ONPG and β-d-galactopyranoside tests, indicating that there are factors that upregulate *bgal-*620.

In this work, we present the concept that AW from cottage cheese has some components that allowed certain LAB to produce high β-galactosidase activity compared with growth in MRS medium. It is very important to stress that *L. helveticus* OSU-PECh-4A characterized in this work grew normally in culture medium but in AW it produced 5 times more β-galactosidase activity. A strain with high β-galactosidase activity may be beneficial in improving the efficiency of lactose hydrolyzation in foods, to decrease the effects of lactose intolerance, and to stimulate the proliferation of beneficial bacteria in the human small intestine. In contrast, the combination of AW and LAB could be useful to reduce the environmental damage caused by AW disposal in wastewater and applied to other industrial waste streams (Mayta-Apaza et al., 2021). It could also have an emerging use in producing beverages enriched in prebiotics such as GOS, taking advantage of the multifunctional enzymatic nature of β-d-galactosidase.
